# Prostate Cancer Screening in Young Men

**DOI:** 10.3390/jpm14080818

**Published:** 2024-07-31

**Authors:** Maxime De Vrieze, Rouvier Al-Monajjed, Matthias Boschheidgen, Peter Albers

**Affiliations:** 1 Division of Personalized Early Detection of Prostate Cancer, German Cancer Research Center (DKFZ), D-69120 Heidelberg, Germany; 2 Department of Urology, Medical Faculty, University Dusseldorf, D-40225 Dusseldorf, Germany; 3 Department of Diagnostic and Interventional Radiology, Medical Faculty, University Dusseldorf, D-40225 Dusseldorf, Germany

**Keywords:** prostate cancer, prostate cancer screening, risk-adapted screening, multiparametric MRI, PSA, baseline PSA

## Abstract

**Background:** Prostate cancer (PCa) screening strategies are being developed and evaluated in several countries. However, most of the evidence regarding PCa screening has been generated in study populations aged 50 and older. **Aims:** This study summarizes findings of a screening trial in younger men and discuss those findings in the context of other screening trials. **Methods:** Non-systematic review. **Results:** Screening of 45-year-old men resulted in a low PCa detection rate. Nonetheless, almost 70% of screen-detected PCa at this age was clinically significant. In young men ISUP GG 1 screen-detected cancers warrant rigorous follow-up. A baseline, midlife prostate-specific antigen (PSA) value at age 45 may safely exclude the vast majority of men from further screening investigations for at least 5 years. At age 45, a confirmatory PSA value reduces the number of subsequent tests almost by half. Sequential magnetic resonance imaging (MRI) as a reflex test subsequent to an elevated PSA ≥ 3 ng/mL needs further investigation in young men. **Conclusions:** Screening in young men needs to be carefully investigated in order to avoid overscreening and overdiagnosis.

## 1. Introduction

A broad consensus regarding the role of population-based screening for prostate cancer (PCa) is currently lacking [1]. The contentious nature of PCa screening stems from uncertainties about the advantages and drawbacks associated with such screening initiatives. Robust evidence indicates a notable reduction in PCa-specific mortality within populations subjected to prostate-specific antigen (PSA) screening [2,3]. Nonetheless, concerns arise due to the harms associated with excessive and invasive diagnostic procedures and overdiagnosis of indolent cancers [4]. Presently, the vast majority of Western countries have opted against organized screening for PCa. In contrast, opportunistic PSA screening in a shared decision-making context, which has unclear effects on oncological outcomes and results in overdiagnosis and health inequities, is prevalent [1,5].

The German “Prostate Cancer Early Detection Study Based on a Baseline PSA Value in Young Men” (PROBASE) is an ongoing randomized clinical trial that investigates risk-adapted PCa screening starting at either 45 or 50 years old [6]. PROBASE provides unique data on a prospectively followed cohort of young men subjected to PCa screening. Although first endpoint-relevant results from the PROBASE trial are expected in about 10 years, valuable knowledge and insights regarding PCa screening in young men can already be derived from PROBASE. The aim of this paper is to summarize and discuss the findings and experiences from PROBASE ten years after starting enrolment.

## 2. What We Have Learned from the PROBASE Trial So Far

### 2.1. Study Protocol

The multicentric PCa screening trial PROBASE tests a risk-adapted PSA-based screening strategy starting at either 45 or 50 years old in a randomized fashion. Study recruitment of more than 46,000 men over a period of 6 years was concluded in 2019. Risk stratification into three self-defined risk groups was performed based solemnly on PSA testing. In accordance with risk-group allocation, participants follow different screening trajectories. Participants deemed at low risk for harboring PCa (PSA < 1.5 ng/mL) are re-invited after a prolonged screening interval of 5 years, whereas participants deemed at higher, intermediate risk for harboring PCa (PSA 1.5–2.99 ng/mL) undergo more frequent, biennial PSA screening. As per the protocol, a confirmed PSA value of ≥3 ng/mL stipulates further diagnostic work-up, including MRI of the prostate and prostatic biopsy. Here, the indication to perform prostate biopsy is independent of MRI findings. If a suspicious lesion (PI-RADS ≥ 3) is detected on MRI, a targeted biopsy of the index lesions is performed, in addition to the standard 12-core systemic biopsy [6].

### 2.2. Results from the First Screening Round at Age 45

In the immediate screening arm at 45 years old, most men (89%) had low PSA values (PROBASE “low risk”) and did not undergo any further interventions for the following 5 years. 10% of the men had PSA values between 1.5 and 3 ng/mL (PROBASE “intermediate risk”) and were scheduled for biennial PSA controls. Only 1% of the men were definitively assigned to the high-risk group, defined as a confirmed PSA level ≥ 3 ng/mL. It is noteworthy that using a confirmatory PSA test two weeks after the initial suspicious PSA test reduced this group by 46% to 186 participants.

Prostate MRI and MRI-guided biopsies were performed in 147 (79%) and 120 (65%) participants, respectively. In total, 48 PCa were screen-detected in the first screening round at age 45 in the immediate screening arm. Most (60%) of these PCa were ISUP GG 2 cancers. Unfavorable intermediate-risk and high-risk disease (ISUP GG ≥ 3) was underrepresented, with only four cases detected. Interestingly, at radical prostatectomy, these cases were all histologically downgraded to ISUP GG 2. Overall, PSA screening of 23,301 men at age 45 showed a clinically significant PCa detection rate of 0.14% [7].

### 2.3. Assessment of the Low-Risk Group

In PROBASE, 89% of the men were allocated to the low-risk group at age 45. In an evaluation of this sub-cohort (PSA < 1.5 ng/mL), 0.45% had a screen-positive PSA test ≥ 3 ng/mL upon re-testing after five years and very few PCa (0.13 cases per 1000-person years) cases were detected as a result. Furthermore, the analysis of different PSA strata showed an exponential increase in both screen-positive PSA tests and screen-detected PCa above this threshold. It was concluded that a baseline PSA level of <1.5 ng/mL at age 45 defines almost 90% of men as having a very low risk of being screen-detected with PCa within five years. Therefore, PROBASE suggested that these men can be safely excluded from re-testing for at least five years [8].

### 2.4. Adherence to the Per-Protocol-Defined Screening Strategy

In PROBASE, participants that were randomized to the immediate screening arm at age 45 showed screening attendance rates to next screening visits at 5 or more years after study enrollment of 70.5% to 79.4%, depending on risk-group allocation and thereby screening intervals. It was observed that attendance at the second screening round strongly predicts attendance at further screening rounds. Men with a family history of PCa or who had a pre-trial history of PSA testing or digital rectal examination (DRE) showed slightly higher participation rates [9].

In the immediate screening arm of PROBASE, a small number of participants (11.2–18.7%) reported having had additional, out-of-protocol PSA tests in between screening rounds. The men that were randomized to the deferred screening arm at age 50 showed a higher rate of so-called PSA “contamination” (25.4%). A subgroup analysis showed that men with a pre-study history of PSA testing (15.8%) and men with a family history of PCa had higher rates of self-initiated PSA testing [9].

In PROBASE, the overall biopsy acceptance rate was 63.6%. Screening PSA value and PI-RADS score seemed to contribute to the participants’ decision to undergo prostate biopsy. The biopsy acceptance was higher for the men who had a screening PSA of ≥4 ng/mL. In spite of biopsy indication being dependent only on a confirmed, elevated PSA value of ≥3 ng/mL, biopsy acceptance was associated with the MRI findings and rose from 59.2% and 72.9% to 92.9% for men with PI-RADS scores of 1–2, 3 and 4–5, respectively [9].

### 2.5. Digital Rectal Examination Is Not a Good PCa Screening Intervention

Using data from PROBASE, the accuracy of DRE in 45-year-olds was prospectively evaluated in a screening setting. The PCa detection rate of DRE was four times lower compared to that of PSA screening. The positive predictive value of DRE as a screening test was extremely low at only 5% in this young cohort. The majority (86%) of all tumors detected by PSA at age 45 were not palpable. Also, when combined with PSA, its performance was notably inadequate and failed to confer any discernible advantages. In conclusion, regarding its low sensitivity and specificity, DRE, especially as a stand-alone test, is not a valid screening modality in 45-year-old men [10].

### 2.6. Quality Control of MRI Is Important in a Screening Setting

MRI expert reading yielded significantly enhanced MRI accuracy in PROBASE. Sensitivity and specificity for PCa detection in a screening setting increased upon reference reading. Notably, on reference reading, the negative predictive value for PI-RADS lesions ≤2 was 100%. However, the proportion of such negative MRI was drastically lower in comparison to older cohorts. In addition, both at local and reference reading, a high proportion of PI-RADS 3 lesions was detected. These results hint at the increased difficulty of interpreting prostate MRI in young men [11].

## 3. Discussion 

### 3.1. Screening of Young Men and the Negative Predictive Value of Low PSA Levels

The exact age at which PCa screening should be started is still debated. Most PCa screening trials included men aged 50 and older [12]. PROBASE set out to show that starting PCa screening at the age of 50, rather than 45, is not harmful. In the first screening round at age 45, only a small number of cancers (0.2%) were screen-detected in PROBASE [7]. In contrast, in the Göteborg- and Stockholm3-trial, that included men older than 50, the PCa detection rate in the first screening round was 2.46% and 3.12%, respectively [13,14]. These results indicate that starting screening from the age of 50 may detect more cancers, including clinically significant cancers according to the current definition (ISUP GG 2 and higher). However, PSA testing at 45 years old allows for the assessment of a baseline, midlife PSA, which could possibly identify those men, who would not profit from extensive screening, and tailor screening interventions in the next decades of life. Recently, PROBASE suggested that men with a PSA < 1.5 ng/mL at age 45 can safely be excluded from further screening interventions for at least 5 years, wherein this interval might be prolonged with extended follow-up. Importantly, no other PCa screening trials have reported on the natural course of PCa development in men with low PSA levels and its consequences for PCa screening.

### 3.2. Compliance with Biopsy Indication

Adherence to a screening program is key to guaranteeing its effectiveness and minimalizing screening-related harms. As PROBASE was conceived in the pre-MRI era, the study protocol prescribes prostate MRI followed by prostate biopsy, where the indication to biopsy is independent of MRI findings, for all participants with a confirmatory screening PSA of ≥3 ng/mL. In PROBASE, a little more than one third of participants did not undergo prostate biopsy as stipulated. However, this is partly a reflection of standard clinical practice, where prostate biopsy indication depends on MRI findings or other clinical parameters such as PSA density. Also, the study population is relatively young. Taking a more current, MRI-guided prostate biopsy indication (PI-RADS ≥ 3) into consideration, PROBASE shows a similar biopsy acceptance rate (80.1%) as its peer trials (Göteborg2: 97.8%; Stockholm3-MRI: 73.3%) [15,16]. In the PROSCREEN trial, a biopsy acceptance rate of 99% was achieved. However, by integrating the 4 K panel based on PSA isoforms after a suspicious PSA test (>3 ng/mL), 30% of men were excluded from further diagnostics and biopsies were only indicated for MRI-positive lesions (PI-RADS ≥ 4) or in cases of high PSA density (≥0.15 ng/L^2^) [17]. Stringent selection of the men to were to undergo prostate biopsy seems to increase willingness to comply therewith.

### 3.3. The Role of MRI in a Sequential Screening Pathway

MRI has been shown to have a high negative predictive value for the detection of clinically significant PCa [18]. In contrast to the exclusive reliance on PSA for PCa screening, the integration of MRI reduces the superfluous biopsy rate and increases specificity [19]. However, it is unclear if evidence favoring a multimodal PSA and MRI approach to PCa screening translates to men below or around 50 years of age. PCa detection by MRI in younger men seems not as straightforward as in older men [11]. Possibly, radiographic characteristics are less distinguishable in earlier tumor stages due to less hyperplasia and atrophy and the presence of diffuse changes in the peripheral zone at T2w-imaging. The complexity of interpreting MRI results from a younger population also raises the question if MRI makes a relevant contribution to the detection of PCa in young men. PROBASE showed the importance of a high-quality MRI reading in a young screening population to guide prostate biopsy indication. The availability of highly qualified radiologists and infrastructure poses a significant challenge to the widescale implementation of MRI in screening. Alternative measures such as centralized readings or the application of artificial intelligence (AI) for MRI assessment may be imperative for the realization of quality control in comprehensive screening programs.

## 4. Conclusions

Findings from PROBASE indicate that evidence from other PCa screening trials should be appraised critically in a young screening population. In the future, PROBASE will be able to address the benefits and drawbacks of starting PCa screening at age 45 versus 50 more in depth.

## Abbreviations

PCa	prostate cancer
PSA	prostate-specific antigen
MRI	magnetic resonance imaging
PI-RADS	Prostate Imaging Reporting and Data System (Version 2.1)
PSAD	prostate-specific antigen density
DRE	digital rectal examination
ISUP GG	International Society of Urological Pathologists Grade Group
AI	artificial intelligence
